# Supplementary figures and images for: Combination treatment of berberine and solid lipid curcumin particles increased cell death and inhibited PI3K/Akt/mTOR pathway of human cultured glioblastoma cells more effectively than did individual treatments
Source: PLoS One. 2019 Dec 16;14(12):e0225660. doi: 10.1371/journal.pone.0225660 (PMC6913937; doi:10.1371/journal.pone.0225660)

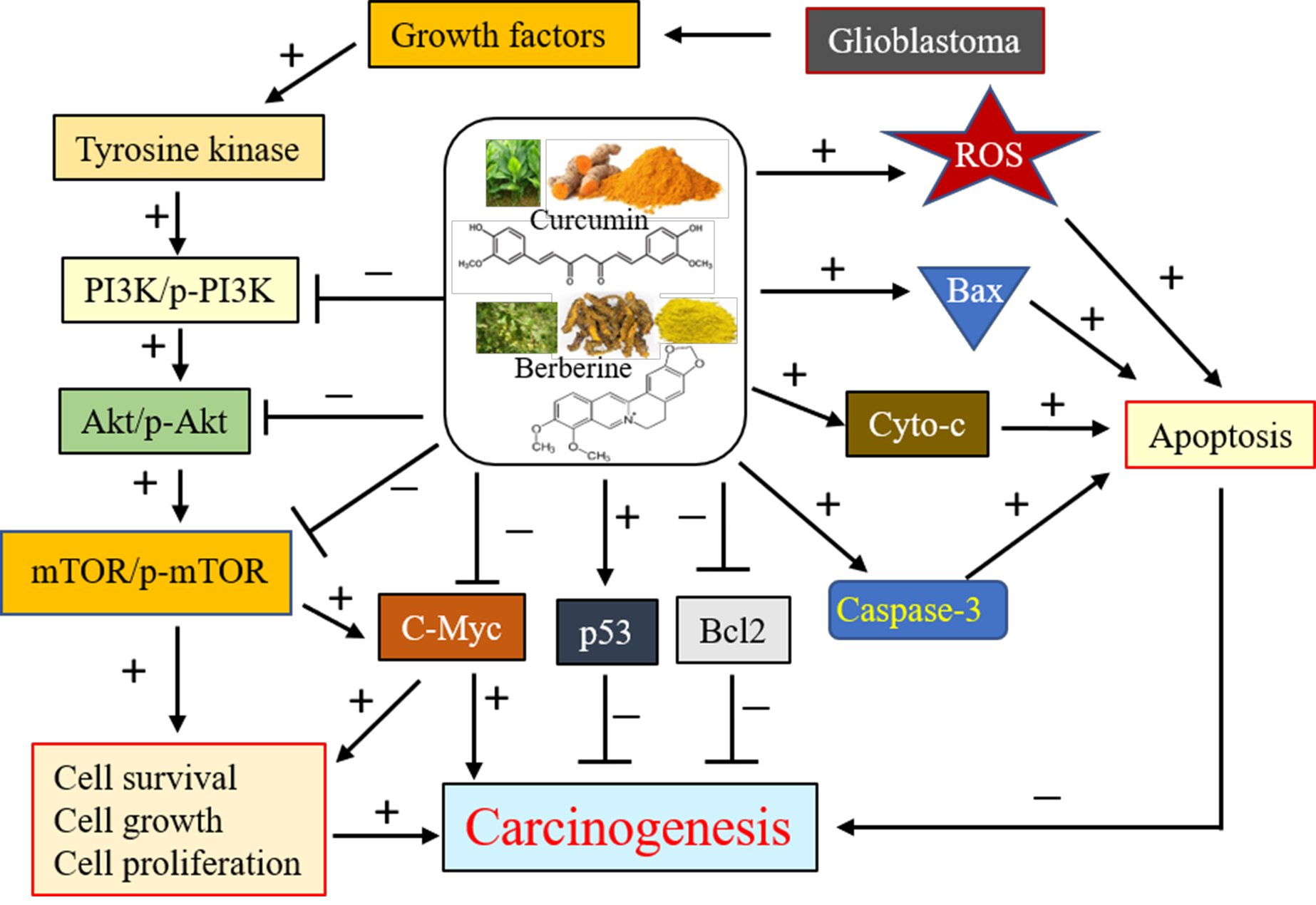

Supplement: S1 Fig — (TIF) [file pone.0225660.s001.tif]
