# Supplementary material for: Combination treatment of berberine and solid lipid curcumin particles increased cell death and inhibited PI3K/Akt/mTOR pathway of human cultured glioblastoma cells more effectively than did individual treatments
Source: PLoS One. 2019 Dec 16;14(12):e0225660. doi: 10.1371/journal.pone.0225660 (PMC6913937; doi:10.1371/journal.pone.0225660)

Fig 5: DNA gel image: The genomic DNA was extracted by QIAmp-DNA Mini Kit (Qiagen) and run with 3% agarose gel

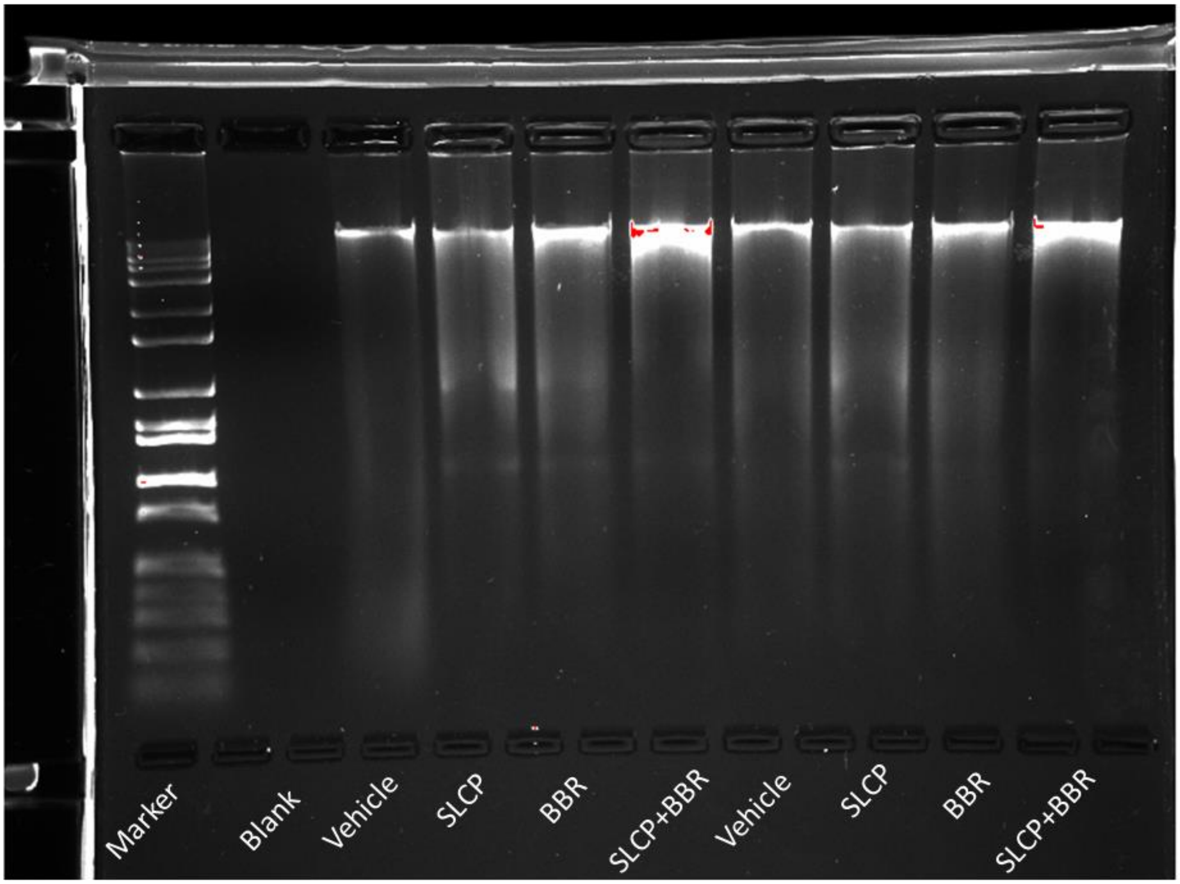

Fig 8A: Western blot of Bax

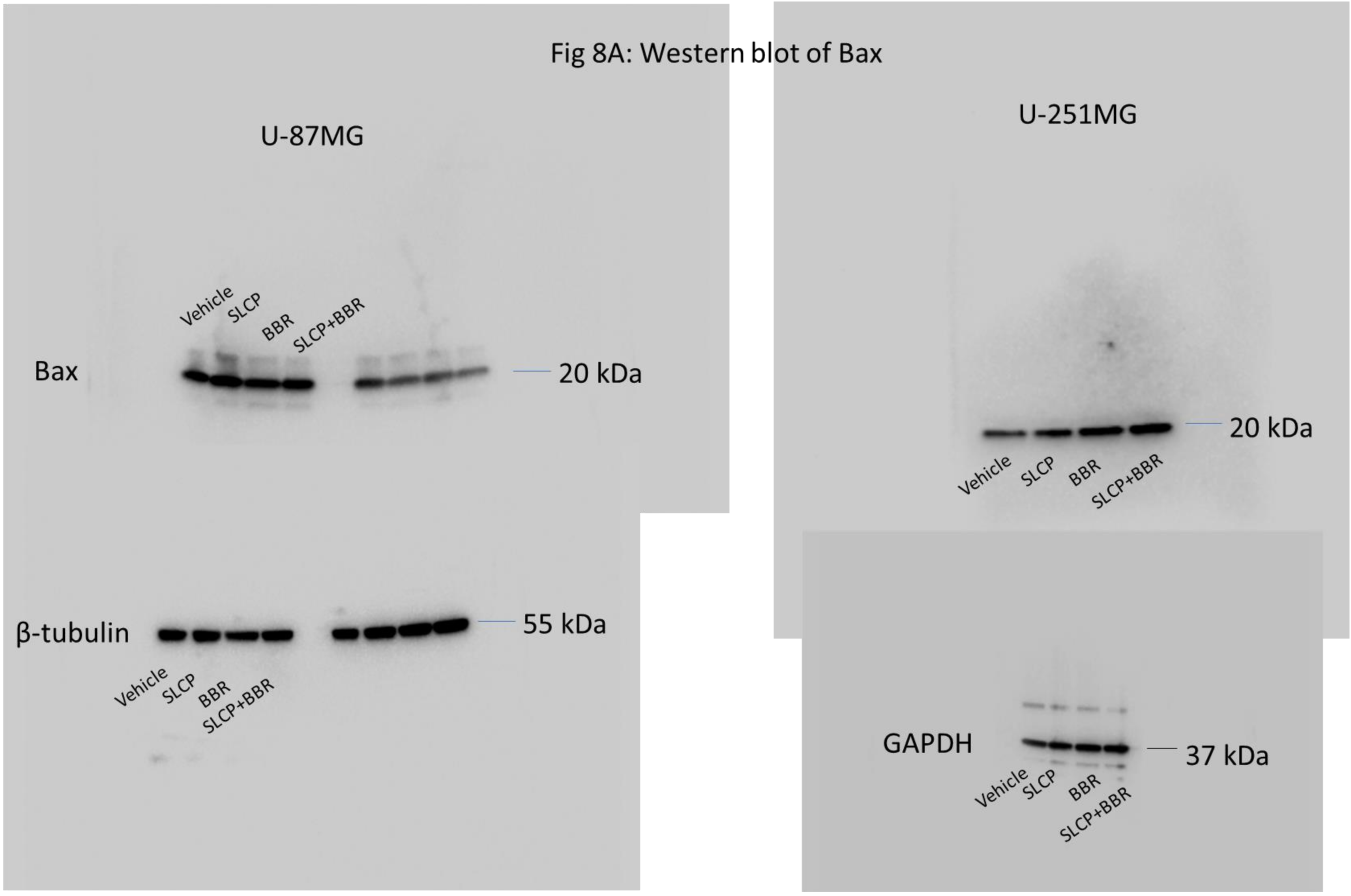

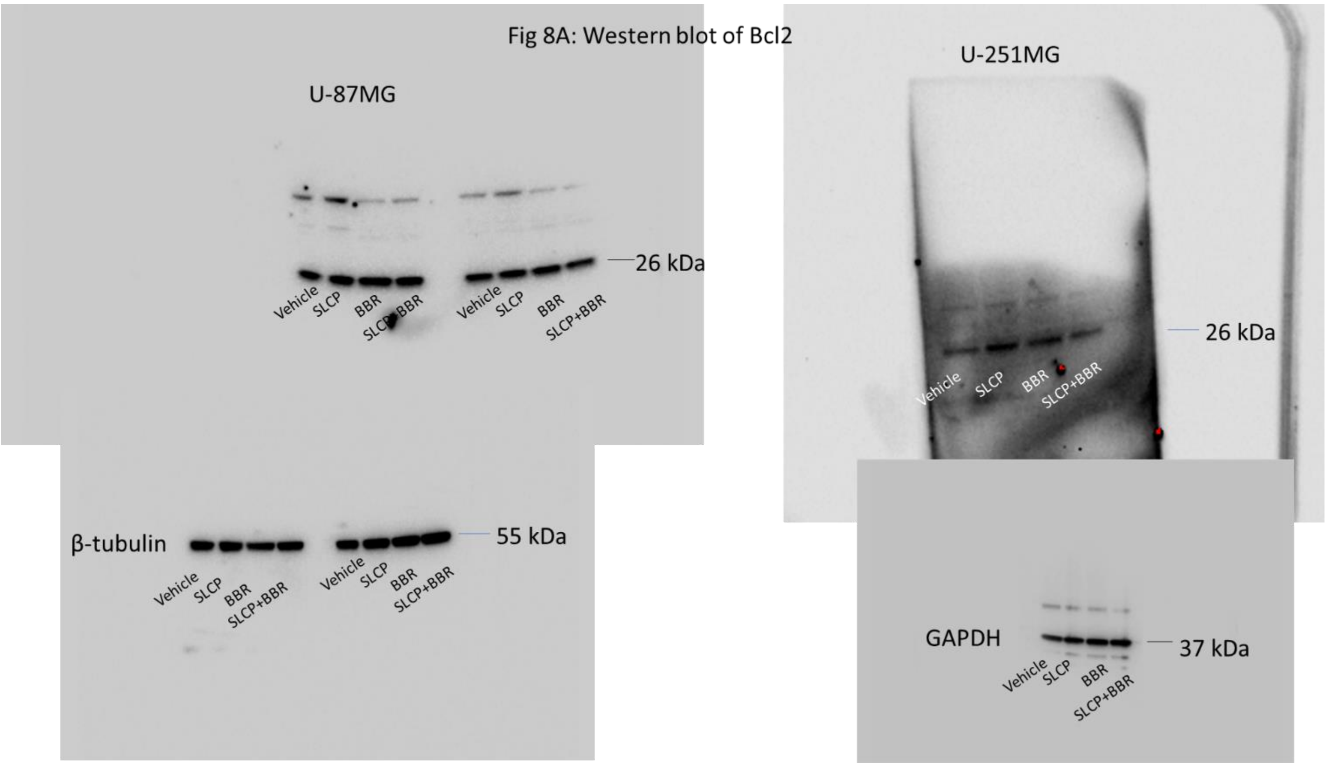

Fig 8A: Western blot of Bcl2

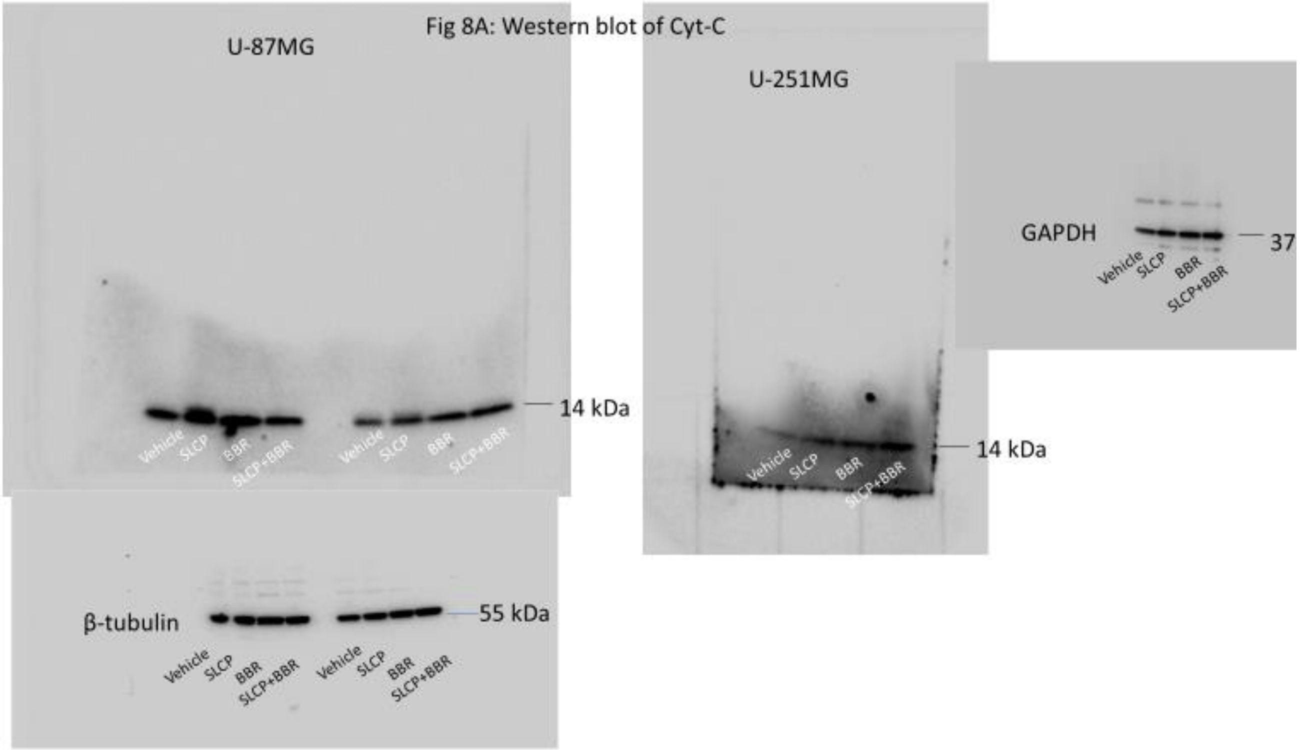

Fig 8A: Western blot of Cyt-C

Fig 8A: Western blot of caspase-3

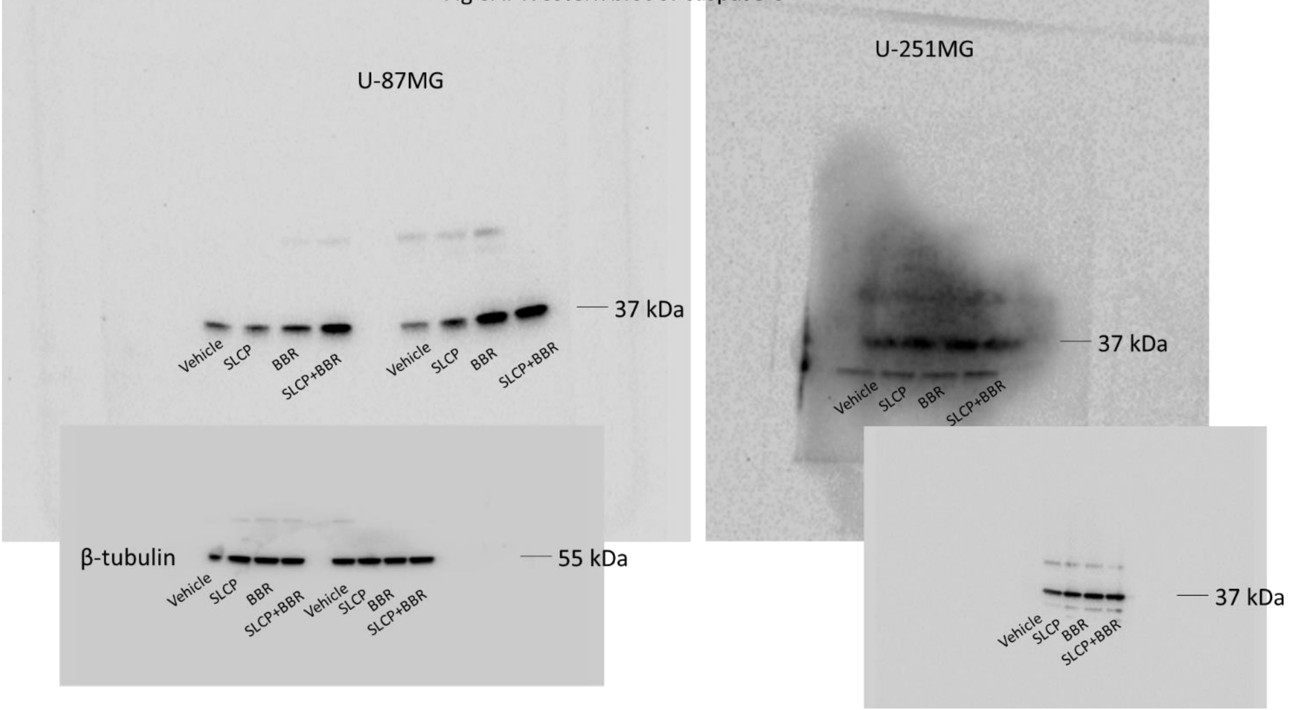

Fig 9A: Western blot of c-Myc

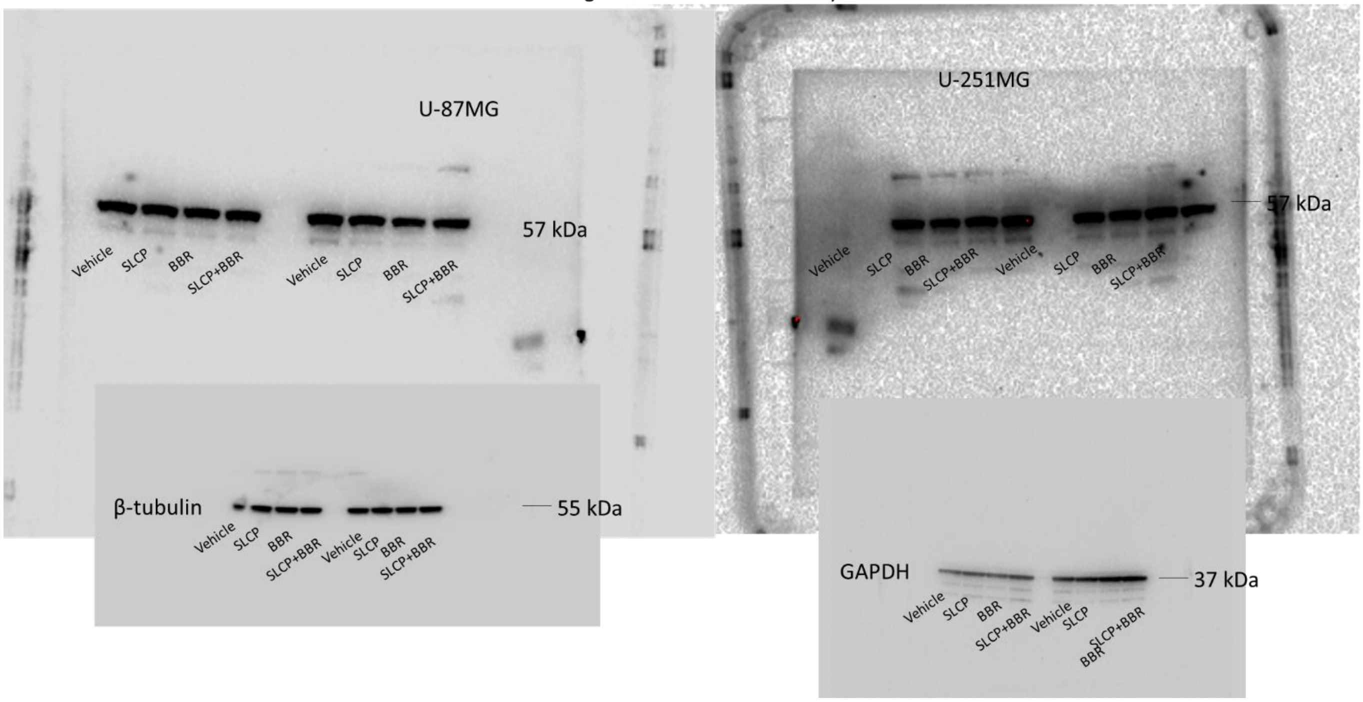

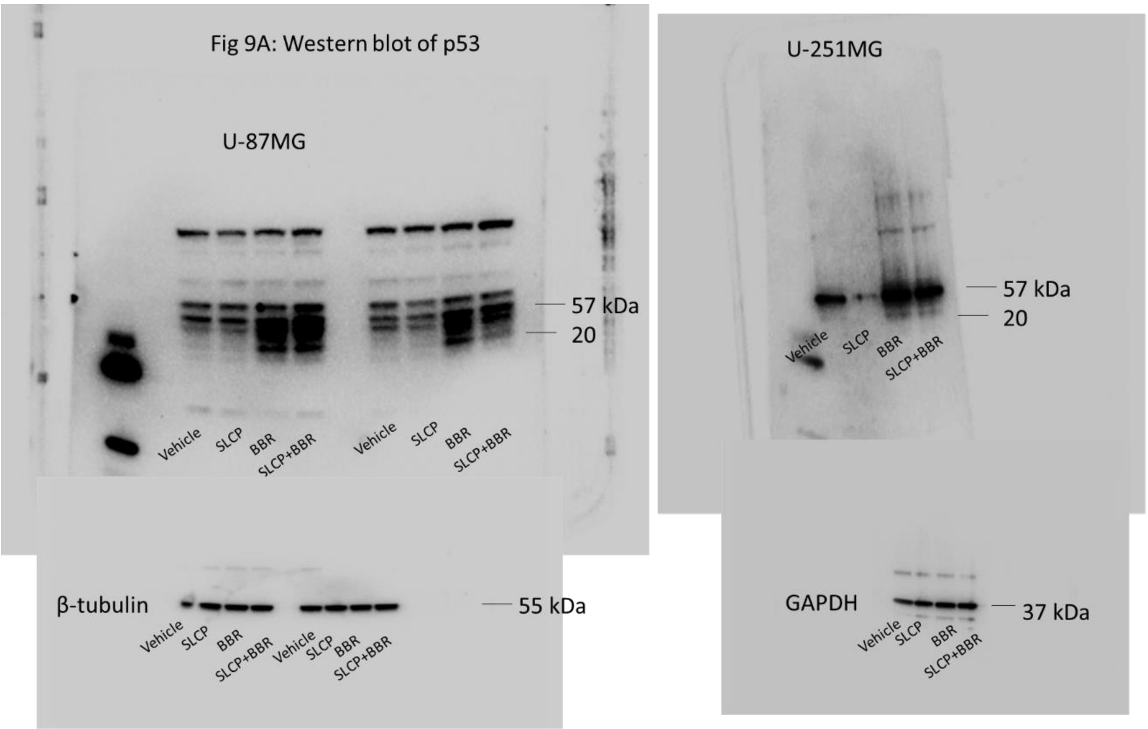
Fig 9A: Western blot of p53
U-87MG
57 kDa
20
Vehicle
SLCP
BBR
SLCP+BBR
Vehicle
SLCP
BBR
SLCP+BBR
β-tubulin
55 kDa
Vehicle
SLCP
BBR
SLCP+BBR
Vehicle
SLCP
BBR
SLCP+BBR
U-251MG
57 kDa
20
Vehicle
SLCP
BBR
SLCP+BBR
GAPDH
37 kDa
Vehicle
SLCP
BBR
SLCP+BBR

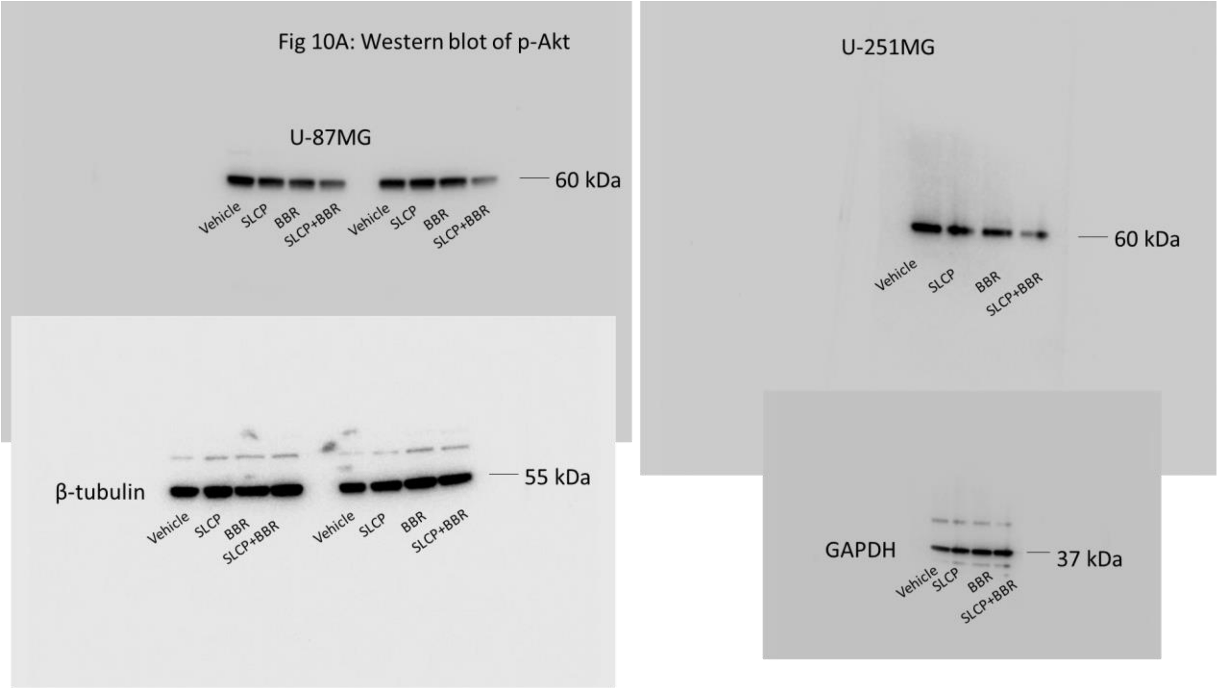
Fig 10A: Western blot of p-Akt
U-87MG
60 kDa
Vehicle
SLCP
BBR
SLCP+BBR
Vehicle
SLCP
BBR
SLCP+BBR
β-tubulin
55 kDa
Vehicle
SLCP
BBR
SLCP+BBR
Vehicle
SLCP
BBR
SLCP+BBR
U-251MG
60 kDa
Vehicle
SLCP
BBR
SLCP+BBR
GAPDH
37 kDa
Vehicle
SLCP
BBR
SLCP+BBR

Fig 10A: Western blot of Akt

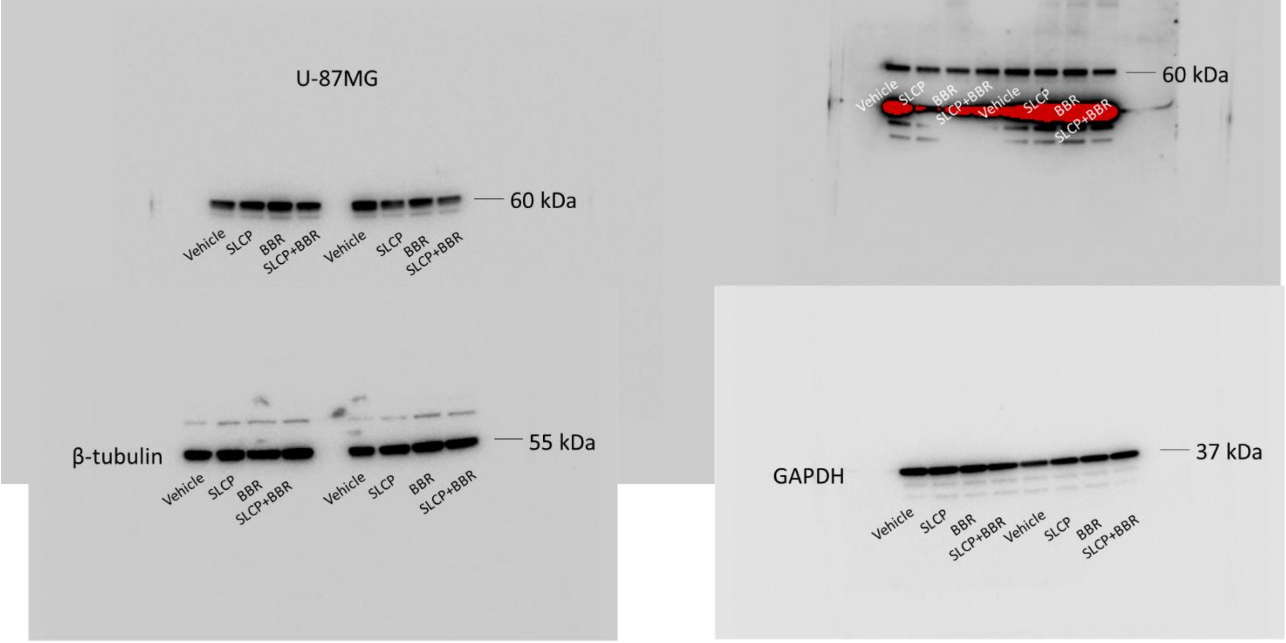

Fig 10A: Western blot of p-PI3K

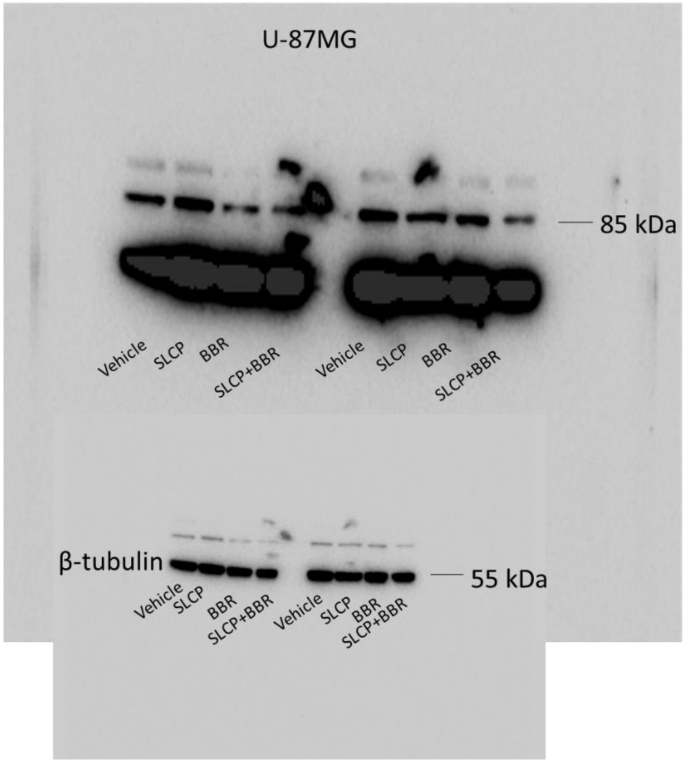

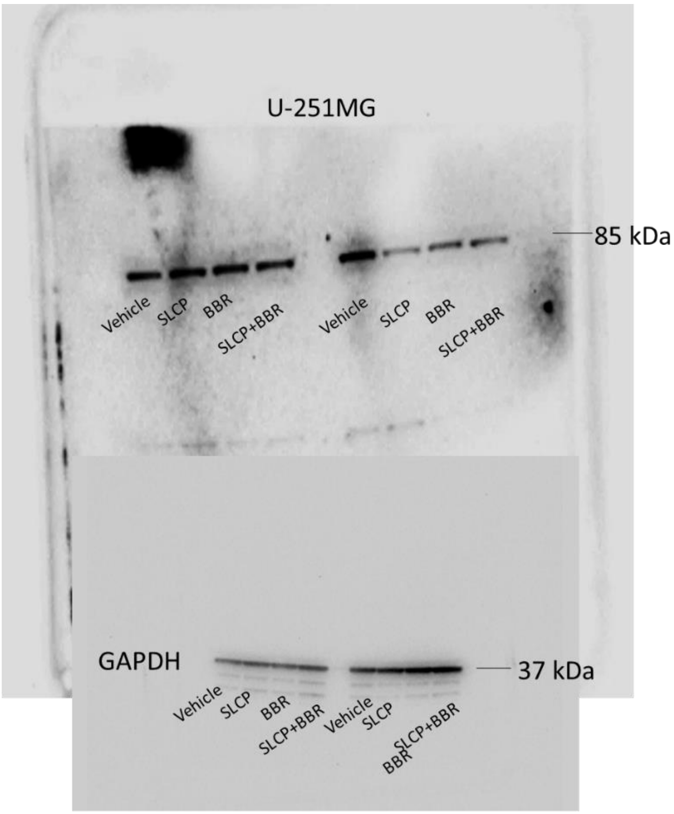

Fig 10A: Western blot of PI3K

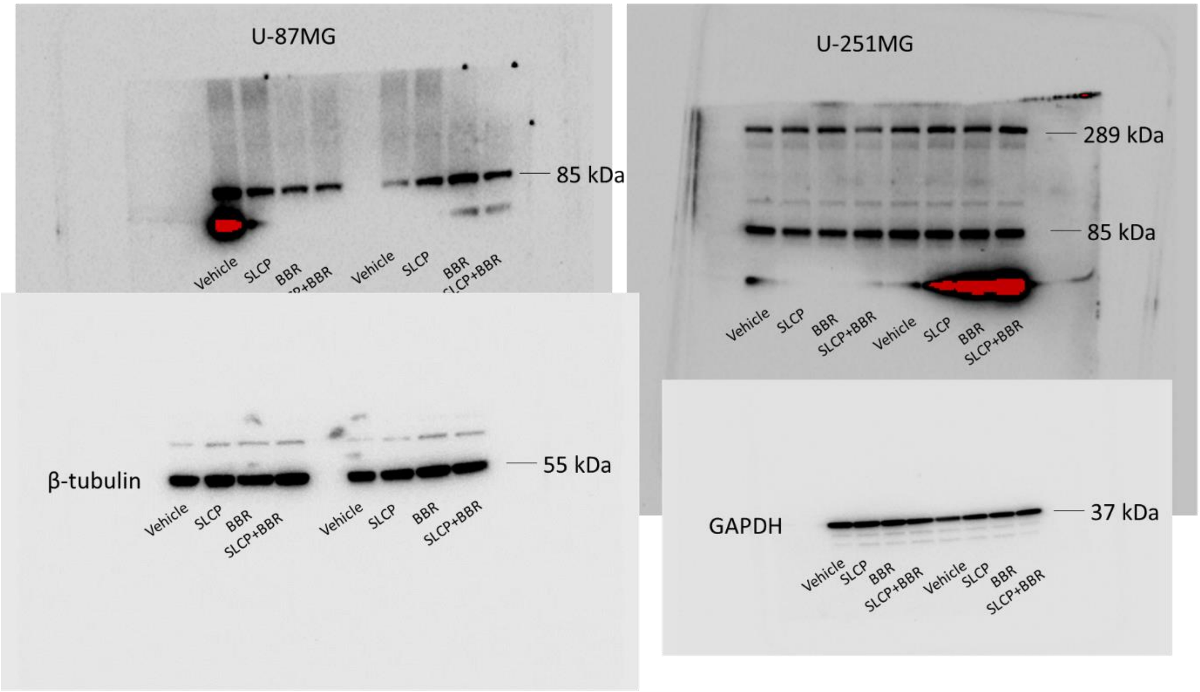

Fig 10A: Western blot of p-mTOR

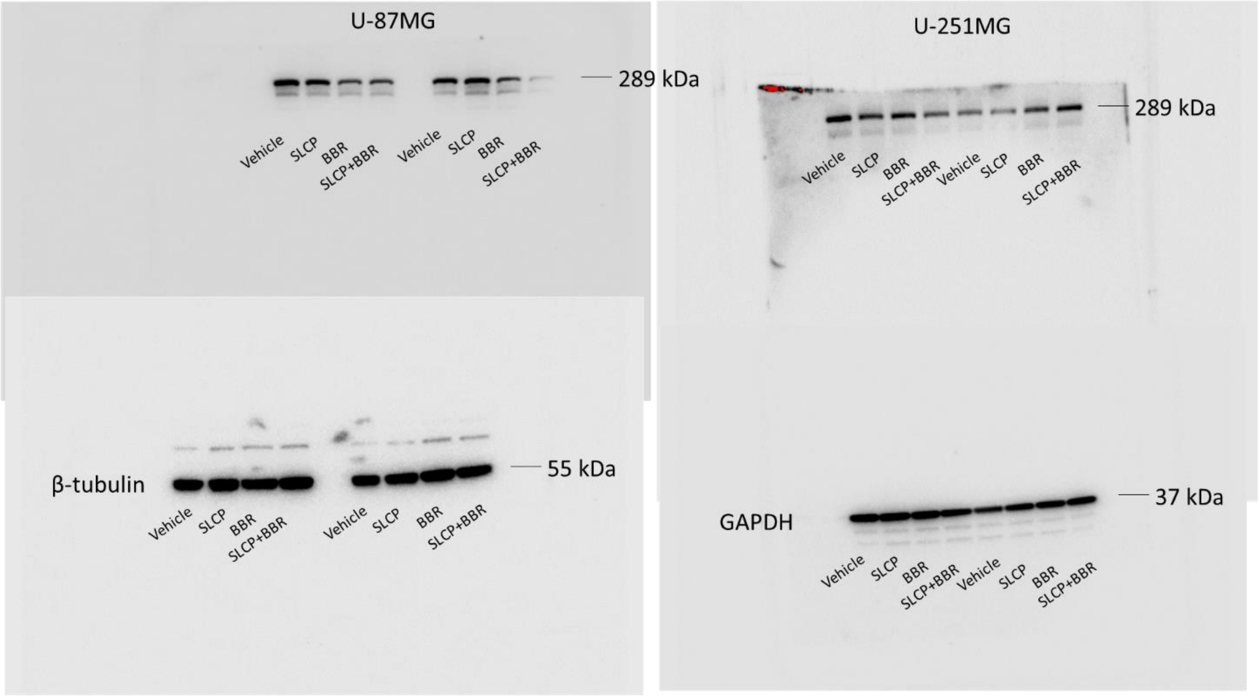

Fig 10A: Western blot of mTOR

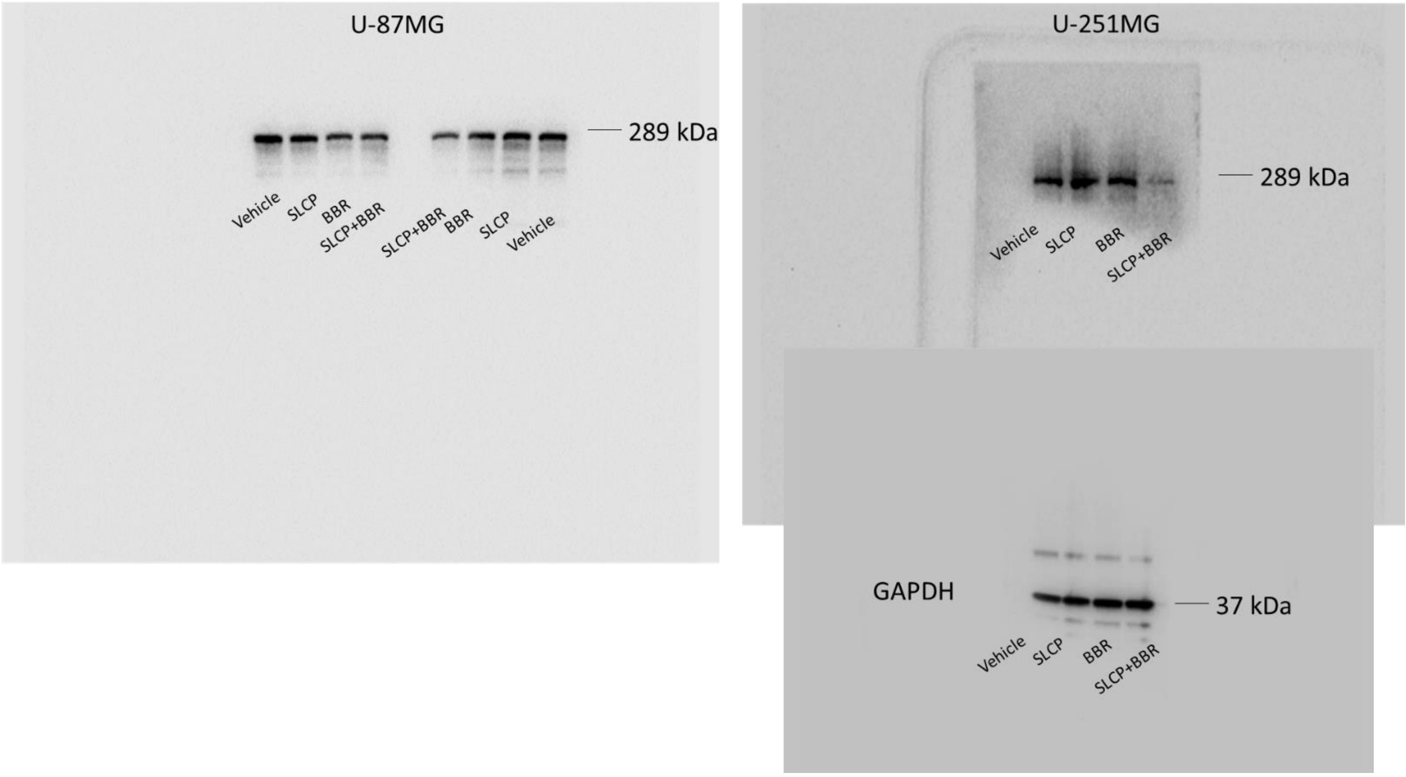

Supplement: S2 Fig — (PDF) [file pone.0225660.s002.pdf]
